# Clinical values of metagenomic next-generation sequencing in patients with severe pneumonia: a systematic review and meta-analysis

**DOI:** 10.3389/fcimb.2023.1106859

**Published:** 2023-04-06

**Authors:** Minjie Lv, Changjun Zhu, Chenghua Zhu, Jing Yao, Lixu Xie, Changwen Zhang, Jianling Huang, Xingran Du, Ganzhu Feng

**Affiliations:** ^1^ Department of Respiratory and Critical Care Medicine, The Second Affiliated Hospital of Nanjing Medical University, Nanjing, Jiangsu, China; ^2^ Department of Infectious Diseases, The Second Affiliated Hospital of Nanjing Medical University, Nanjing, Jiangsu, China; ^3^ Department of Respiratory and Critical Care Medicine, The Affiliated Jiangning Hospital of Nanjing Medical University, Nanjing, Jiangsu, China

**Keywords:** metagenomic next-generation sequencing, severe pneumonia, diagnosis, prognosis, conventional methods

## Abstract

**Background:**

Clinical values of metagenomic next-generation sequencing (mNGS) in patients with severe pneumonia remain controversial. Therefore, we conduct this meta-analysis to evaluate the diagnostic performance of mNGS for pathogen detection and its role in the prognosis of severe pneumonia.

**Methods:**

We systematically searched the literature published in PubMed, Embase, Cochrane Library, Web of Science, Clinical Trials.gov, CNKI, Wanfang Data, and CBM from the inception to the 28th September 2022. Relevant trials comparing mNGS with conventional methods applied to patients with severe pneumonia were included. The primary outcomes of this study were the pathogen-positive rate, the 28-day mortality, and the 90-day mortality; secondary outcomes included the duration of mechanical ventilation, the length of hospital stay, and the length of stay in the ICU.

**Results:**

Totally, 24 publications with 3220 patients met the inclusion criteria and were enrolled in this study. Compared with conventional methods (45.78%, 705/1540), mNGS (80.48%, 1233/1532) significantly increased the positive rate of pathogen detection [*OR* = 6.81, 95% *CI* (4.59, 10.11, *P* < 0.001]. The pooled 28-day and 90-day mortality in mNGS group were 15.08% (38/252) and 22.36% (36/161), respectively, which were significantly lower than those in conventional methods group 33.05% (117/354) [*OR* = 0.35, 95% *CI* (0.23, 0.55), *P* < 0.001, *I^2^* = 0%] and 43.43%(109/251) [*OR* = 0.34, 95% *CI* (0.21, 0.54), *P* < 0.001]. Meanwhile, adjusted treatment based on the results of mNGS shortened the length of hospital stay [MD = -2.76, 95% *CI* (− 3.56, − 1.96), P < 0.001] and the length of stay in ICU [*MD* = -4.11, 95% *CI* (− 5.35, − 2.87), *P* < 0.001].

**Conclusion:**

The pathogen detection positive rate of mNGS was much higher than that of conventional methods. Adjusted treatment based on mNGS results can reduce the 28-day and 90-day mortality of patients with severe pneumonia, and shorten the length of hospital and ICU stay. Therefore, mNGS advised to be applied to severe pneumonia patients as early as possible in addition to conventional methods to improve the prognosis and reduce the length of hospital stay.

## Introduction

Severe pneumonia is a common critical illness with an increasing morbidity and mortality, patients with this disease often require admission to intensive care unit (ICU) (Kalil et al., 2016; Metlay et al., 2019; Zhou et al., 2021). Despite significant advances in its etiological investigation and antimicrobial therapy, severe pneumonia remains the leading cause of death among infectious diseases worldwide (De Pascale et al., 2011). There is no denying that early, rapid, and accurate pathogenic diagnosis is crucial in guiding promt antibiotic treatment, thus improving prognosis and reducing fatality (Cillóniz et al., 2021).

Traditionally, clinicians select antibiotics empirically and then adjust treatment based on the results of conventional methods. However, microbiological culture-based tests do not meet clinical needs due to their time-consuming nature, low sensitivity, lack of diagnostic tests for rare pathogens and vulnerability to external influences (Torres et al., 2021). Inspiringly, metagenomic next-generation sequencing (mNGS), a new pathogen detection technique with high efficiency, broad pathogen spectrum and increased sensitivity, has been widely used in clinic gradually (Gu et al., 2019). mNGS theoretically performs unbiased and detailed high-throughput sequencing of the total DNA or RNA content of almost all known pathogens, including bacteria, fungi, viruses, mycobacterium tuberculosis, parasites and atypical pathogens, and then compares the obtained sequence information with databases (Chiu and Miller, 2019). Several case reports and clinical trials have demonstrated the great value of mNGS in the pathogenic diagnosis of severe and complex cases, including rare pathogens, mixed infections (Wang et al., 2019) and infectious diseases presenting with atypical symptoms, such as Chlamydia psittaci (Li et al., 2021), Chlamydia abortus (Zhu et al., 2022), leptospirosis presenting as severe alveolar hemorrhage (Chen M. et al., 2021) and so on. Several studies (Huang et al., 2020; Chen S. et al., 2022; Wu et al., 2022) have revealed that the sensitivity and specificity of mNGS detection are markedly superior than that of conventional methods.

However, the results of studies on the diagnostic and prognositic values of mNGS on severe pneumonia are controversial (Xie et al., 2019; Zhang et al., 2020; Xi et al., 2022). In addition, to our knowledge, there is no relevant systematic review and meta-analysis to provide a higher level of evidence on the clinical values of mNGS results on severe pneumonia. Therefore, we performed this meta-analysis to evaluate and compare mNGS and conventional methods on the diagnostic performance and prognostic impact of severe pneumonia.

## Methods

We strictly followed the standards of the Preferred Reporting Items for Systematic Reviews and Meta-Analyses (PRISMA) (http://www.prisma-statement.org/) in reporting the findings of this review. The protocol for this study was registered in PROSPERO (CRD42022325564).

### Search strategy and data sources

PubMed, Embase, Cochrane Library, Web of Science, Clinical Trials.gov, CNKI, Wanfang DATA and CBM were searched from inception to 28th September 2022. Search terms included the following: (“Next generation sequencing” OR “Metagenomic next generation sequencing” OR “NGS” OR “mNGS”) AND (“severe pneumonia” OR “serious pneumonia” OR “severe respiratory infection” OR “severe lung infection” OR “severe community-acquired pneumonia” OR “severe hospital-acquired pneumonia”). Researchers manually scanned the references of all retrieved articles and other relevant publications for additional articles. The exhaustive search strategy is reported in **Table S1**.

### Eligibility criteria and study selection

Two authors (Lv M and Zhu C) screened the relevant literature independently and then checked the title and abstract of each retrieved article to decide which required further assessment. The full text of potentially eligible records was retrieved, reviewed and eligible studies were included. When there were disagreements, Lv M, Zhu C and Du X discussed thoroughly to reach an agreement. Severe pneumonia was defined in patients with either one major criterion or at least three minor criteria of the Infectious Diseases Society of America (IDSA)/American Thoracic Society (ATS) criteria (Metlay et al., 2019).

The primary outcomes were pathogen detection positive rate, 28-day mortality, and 90-day mortality. Secondary outcomes included duration of mechanical ventilation, length of hospital stay, and length of stay in the ICU. The eligibility criteria were as follows: (1) patients with severe pneumonia; (2) participants above 18 years old; (3) reports comparing the pathogen detection positive rate or prognosis outcomes of mNGS with conventional methods. Articles were excluded based on the following criteria: (1) reviews, letters, case report, or case series with < 10 patients; (2) enrolled patients did not suffer from severe pneumonia; (3) not provide data on pathogen-detection positive rate, mortality or prognostic factors; (4) studies not compare positive rate, mortality or prognostic factors between mNGS group and conventional methods group; (5) low quality or can not obtain relevant data.

### Qualitative assessment and data extraction

The quality of each study was independently evaluated by two authors (Lv M and Zhu C) using the Newcastle-Ottawa scale. Two authors (Lv M and Zhu C) independently extracted data with a customized data extraction form and assessed the risk of bias. The data extraction form included the following detailed information: (1) references and publication date; (2) type of research; (3) sample types; (4) mean age; (5) gender; (6) the initial value of the diagnostic performance indicators; (7) 28-day mortality; (8) 90-day mortality; (9) duration of mechanical ventilation; (10) length of hospital stay; and (11) length of stay in ICU.

### Meta-analysis and statistical methods

Meta-analysis was used to synthesize the outcome measure estimates. Data analyses were performed by Review Manager 5.4. A funnel plot was applied to check for publication bias, and *I^2^* was applied to estimate the total variation attributed to heterogeneity among studies. Sensitivity analysis was performed by Stata 15. For dichotomous variables, odds ratios (*ORs*) were used for statistical calculations, whereas mean differences (*MDs*) were used for continuous variables. For *I^2^* ≥ 50%, the random effect model of the restricted maximum likelihood probability method is used. Otherwise, the fixed effect model of the reverse variance method is used.

## Results

### Study selection process and data extraction

**Figure 1:**
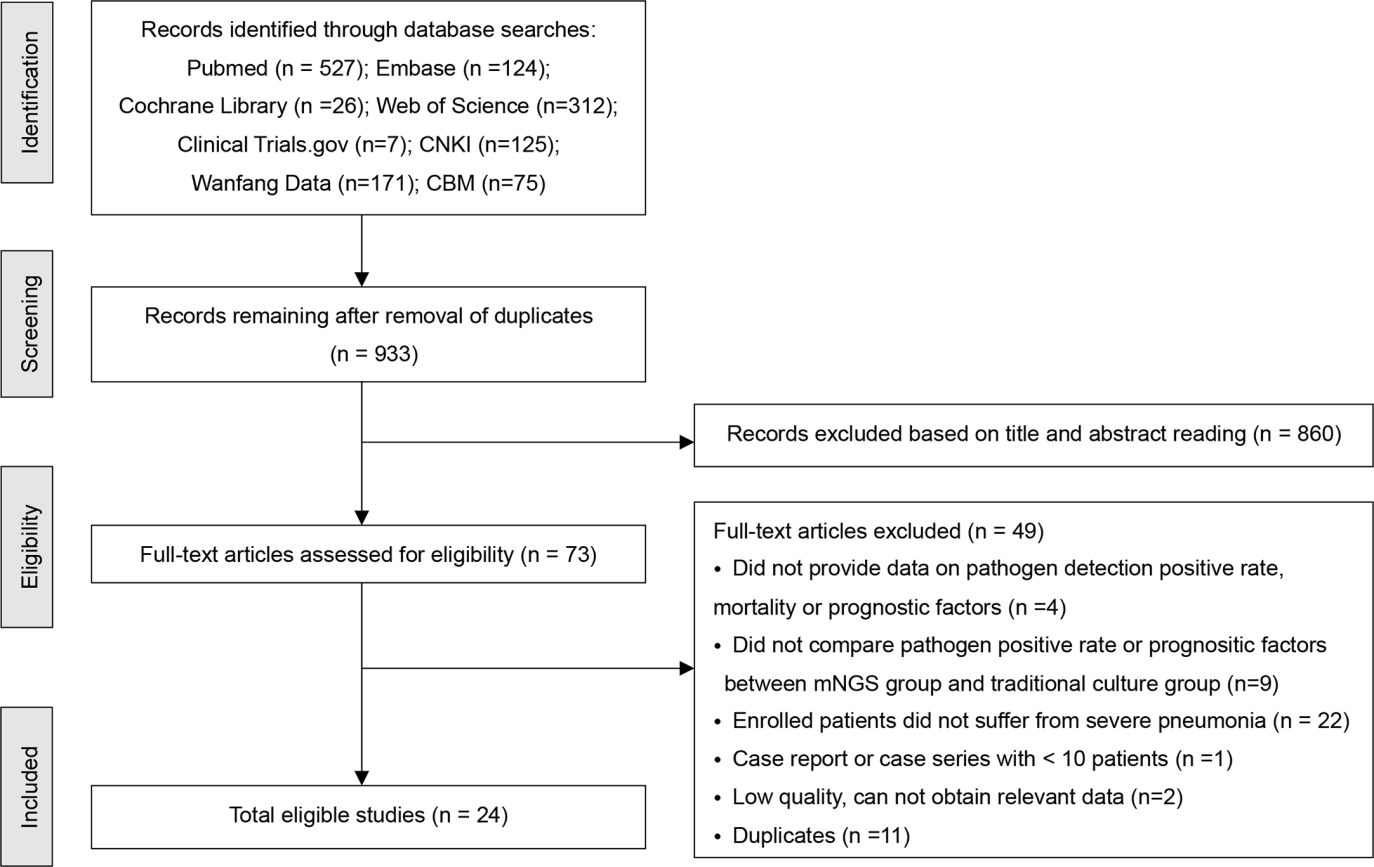
The flow diagram of included studies.

The search identified 1367 records, among these 434 were removed as duplicates. After screening titles and abstracts, 860 were found to be ineligible. Overall, 73 full-text articles were assessed for eligibility. Ultimately, 24 studies met the inclusion criteria (Wu, 2019; Xie et al., 2019; Zhuo et al., 2019; Chen, 2020; Fan, 2020; Pan, 2020; Pan and Lv, 2020; Ren et al., 2020; Song et al., 2020; Sun et al., 2020; Wu et al., 2020; Zhang et al., 2020; Chen J. et al., 2021; Huang, 2021; Lu et al., 2021; Ma et al., 2021; Xu, 2021; Zhu and Cao, 2021; Chen Y. et al., 2022; Huang, 2022; Liu et al., 2022; Tan et al., 2022; Zhang et al., 2022; Zhou and Yang, 2022). The detailed literature retrieval and screening process is shown in **Figure 1**.

### Characteristics of the included studies

The characteristics of the eligible studies are presented in **Table 1**. Totally, 24 studies with 3220 patients were enrolled. Nineteen (Wu, 2019; Xie et al., 2019; Chen, 2020; Fan, 2020; Pan, 2020; Pan and Lv, 2020; Ren et al., 2020; Song et al., 2020; Sun et al., 2020; Zhang et al., 2020; Chen J. et al., 2021; Lu et al., 2021; Ma et al., 2021; Xu, 2021; Chen Y et al., 2022; Huang, 2022; Liu et al., 2022; Tan et al., 2022; Zhou and Yang, 2022) studies were retrospective and five (Zhuo et al., 2019; Wu et al., 2020; Huang, 2021; Zhu and Cao, 2021; Zhang et al., 2022) were prospective. Eighteen studies included patients with severe pneumonia and six studies included severe pneumonia patients with other complications, such as immunosuppression (Ma et al., 2021), immunodeficiency (Lu et al., 2021), bloodstream infection (Chen J. et al., 2021), acute respiratory distress syndrome (ARDS) (Zhang et al., 2020), after renal transplantation (Zhuo et al., 2019) and autoimmune diseases (Wu, 2019). The sample types collected mainly included blood, sputum, or bronchoalveolar lavage fluid (BALF). Among them, 23 studies (95.8%), 6 studies (25.0%), and 4 studies (16.7%) reported pathogen detection positive rate, 28-day mortality, and 90-day mortality, respectively. Some studies assessed detection positive rate of bacteria, fungi, virus and other pathogens respectively, which are listed in **Table S2**. Useable data for the duration of mechanical ventilation, length of hospital stay and length of stay in ICU were provided in 5 studies (20.8%), 6 studies (25.0%), and 4 studies (16.7%). The mean study quality score of the studies was 6.38 (SD=0.88) out of 9 on the Newcastle-Ottawa Scale, representing moderate to high methodological quality. A detailed quality assessment is presented in **Table S3**.

**Table 1:** The characteristics of the included preclinical studies.

Author	Year	Type of research	Disease Types	Total, mNGS, control(N)	Sample Types	Detection Positive rate (%)		Mean Age, [mNGS, control (years)]		Proportion of men, [mNGS, control, N(%)]	
						mNGS	Control	mNGS	Control	mNGS	Control
**Anbing Zhang**	2022	Prospective	Severe pneumonia	112,56,56	BALF	92.9	51.8	62.57	60.91	26(46.4)	29(51.8)
**Youlian Chen**	2022	Retrospective	Severe pneumonia	116,58,58	BALF	91.4	32.8	62.50	62.50	30(51.7)	30(51.7)
**Xiaolian Zhou**	2022	Retrospective	Severe pneumonia	140,70,70	BALF	98.0	69.4	69.20	69.20	51(72.9)	51(72.9)
**Sujun Huang**	2022	Retrospective	Severe pneumonia	60,30,30	blood, sputum, or BALF	90.0	56.7	69.72	69.80	18(60.0)	14(46.7)
**Wenwen Tan**	2022	Retrospective	Severe pneumonia	320,160,160	BALF	51.9	46.9	75.41	75.41	95(59.4)	95(59.4)
**Hanying Liu**	2022	Retrospective	Severe community- acquired pneumonia	346,173,173	sputum or BALF	64.0	28.0	60.00	64.00	126(72.8)	106(61.3)
**Chunyan Huang**	2021	Prospective	Severe pneumonia	60,30,30	blood or BALF	73.3	36.7	56.43	56.83	17(56.7)	16(53.3)
**Fuyao Zhu**	2021	Prospective	Severe pneumonia	48,24,24	blood, sputum, or BALF	91.7	66.7	70.75	74.67	13(54.2)	14(58.3)
**Xiaolong Ma**	2021	Retrospective	Immunosuppression complicated with severe pneumonia	60,30,30	BALF	83.3	40.0	54.70	55.30	21(70.0)	23(76.7)
**Jiancong Lu**	2021	Retrospective	Immunodeficiency with severe pneumonia	152,76,76	BALF	89.5	51.3	38.46	38.46	44(57.9)	44(57.9)
**Jinlian Chen**	2021	Retrospective	Severe pneumonia with bloodstream infection	40,20,20	blood or BALF	85.0	50.0	53.05	53.05	12(60.0)	12(60.0)
**Yuhui Xu**	2021	Retrospective	Severe pneumonia	220,110,110	blood, sputum, or BALF	74.5	45.5	56.89	56.89	76(69.1)	76(69.1)
**Peng Zhang**	2020	Retrospective	ARDS caused by severe pneumonia	95,42,53	blood, sputum, or BALF	91.1	62.2	NR	NR	31(73.8)	38 (71.7)
**Chanyuan Pan**	2020	Retrospective	Severe pneumonia	148,115,33	blood, sputum, or BALF	90.4	47.8	61.28	65.64	83(72.2)	26(78.8)
**Di Ren**	2020	Retrospective	Severe pulmonary infection	87,43,44	blood or BALF	69.7	36.2	56.05	57.57	29(67.4)	27(61.4)
**C. Song**	2020	Retrospective	Severe pneumonia	148,74,74	NR	NR	NR	NR	NR	NR	NR
**Xiaodong Wu**	2020	Prospective	Severe community- acquired pneumonia	658,329,329	BALF	90.3	39.5	64.00	64.00	207(62.9)	207(62.9)
**Ling Chen**	2020	Retrospective	Severe pneumonia	56,28,28	BALF	75.6	32.1	54.82	54.82	21(75.0)	21(75.0)
**Xinyuan Fan**	2020	Retrospective	Severe pneumonia	40,20,20	BALF	70.0	35.0	58.70	58.70	NR	NR
**Guoxian Sun**	2020	Retrospective	Severe pneumonia	16,8,8	BALF	100.0	37.5	62.50	62.50	5(62.5)	5(62.5)
**Chunxi Pan**	2020	Retrospective	Severe pneumonia	52,26,26	blood or BALF	92.3	42.3	45.27	45.27	21(80.8)	21(80.8)
**Huichang Zhuo**	2019	Prospective	Severe pneumonia after renal transplantation	38,15,23	BALF	100.0	69.6	40.90	45.30	11(73.3)	18(78.2)
**Jing Wu**	2019	Retrospective	Autoimmune diseases with severe pneumonia	36,18,18	BALF	83.3	33.3	55.00	55.00	7(41.1)	7(41.1)
**Yun Xie**	2018	Retrospective	Severe pneumonia	178,48,130	blood, sputum, or BALF	97.9	75.4	NR	NR	NR	NR

BALF, bronchoalveolar lavage fluid; NR, not reported.

### Meta-analysis: mNGS versus conventional methods

#### Pathogen detection positive rate

**Figure 2:**
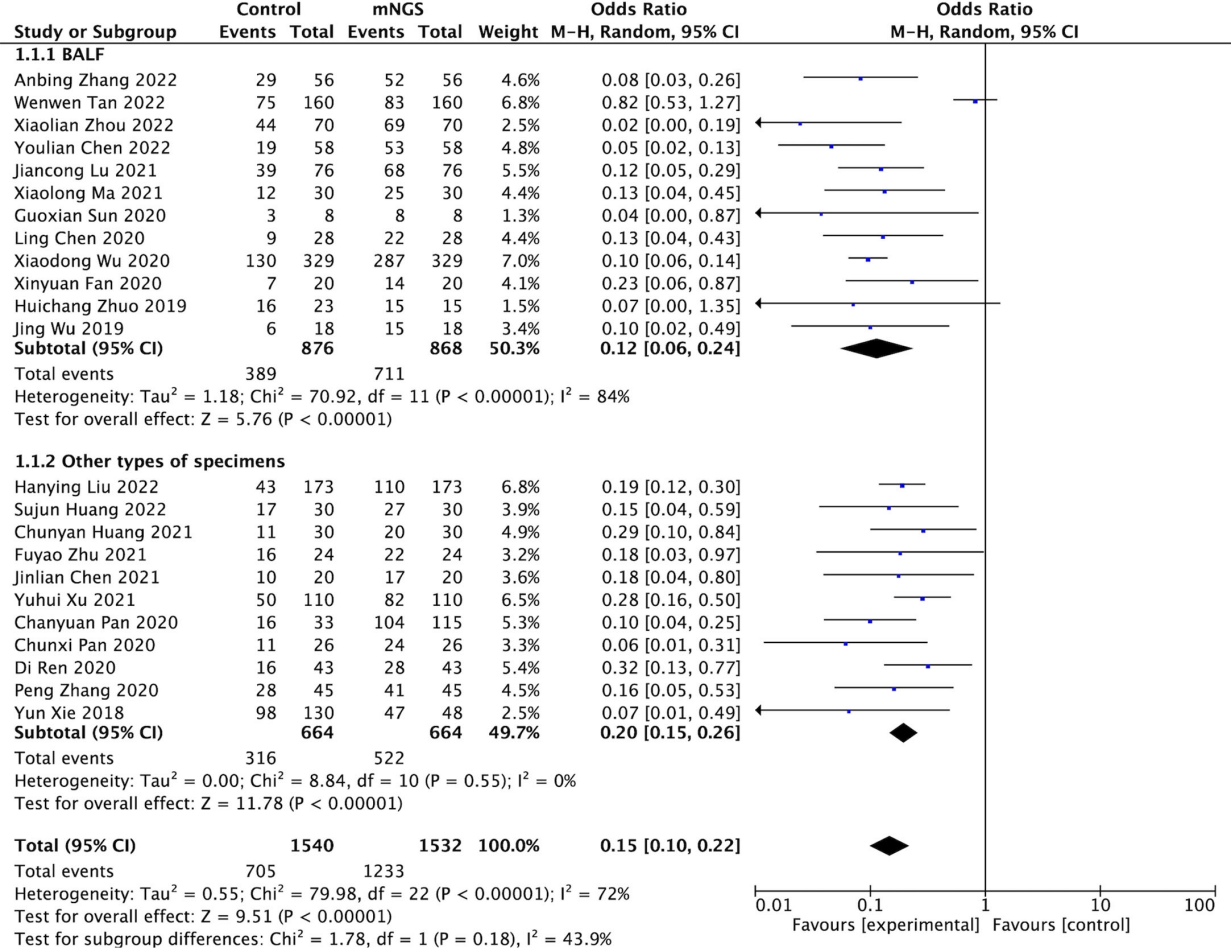
Comparison of pathogen detection positive rate between mNGS and conventional methods group.

**Figure 3:**
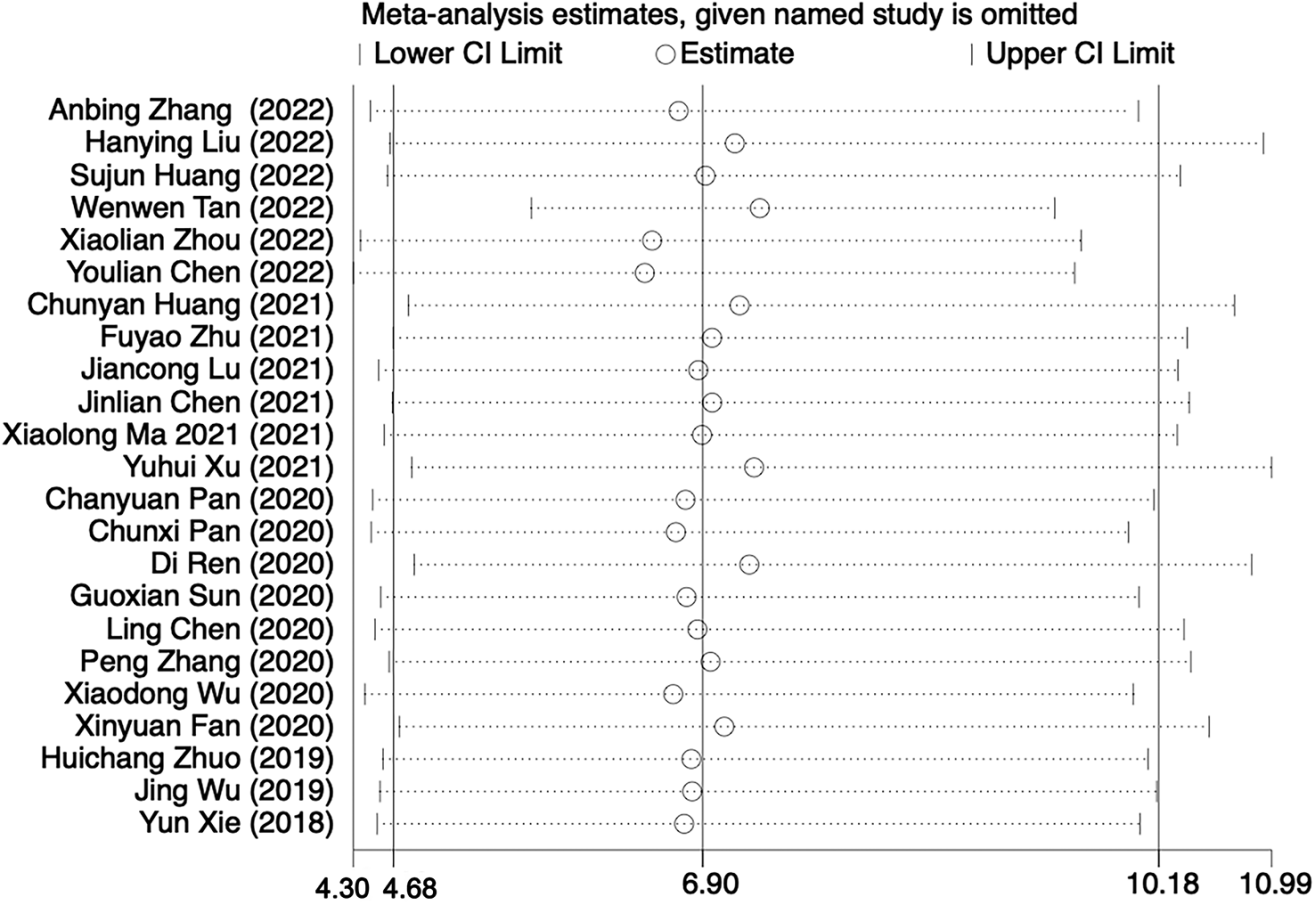
Sensitivity analysis of mNGS and conventional methods group.

**Figure 4:**
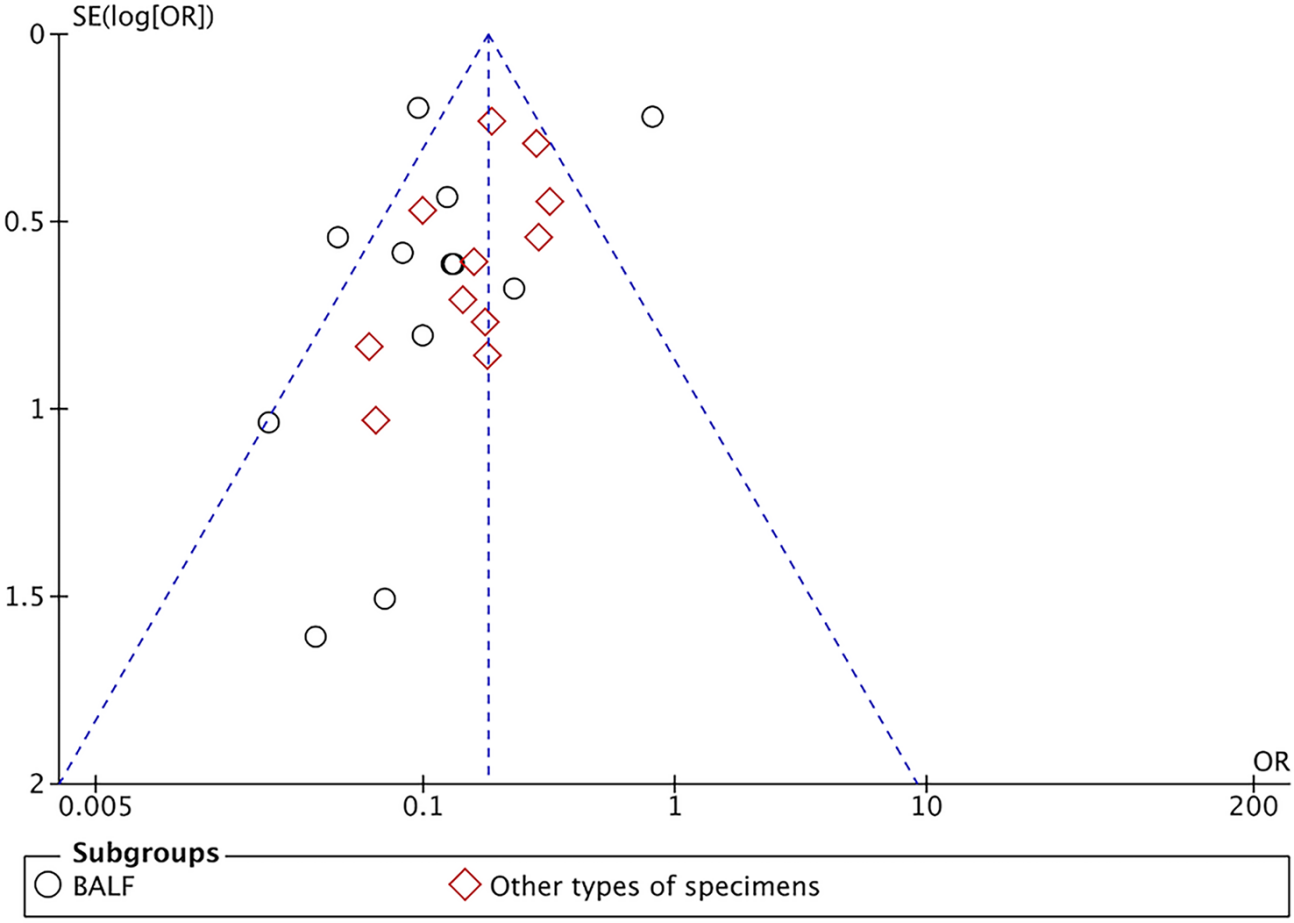
Funnel plot of pathogen detection positive rate between mNGS and conventional methods group.

A total of 3072 cases in 23 studies were pooled for assessing pathogen detection positive rate. The random-effect model was applied given the heterogeneity test indicated moderate heterogeneity (*I^2^* > 50%, *P* < 0.001). We introduced subgroups including BALF and other types of specimens group. The meta-analysis results showed that compared with the conventional methods group (45.78%, 705/1540), mNGS group (80.48%, 1233/1532) significantly increased the pathogen detection positive rate [*OR* = 6.81, 95% *CI* (4.59, 10.11), *P* < 0.001 (**Figure 2**). Sensitivity analysis (**Figure 3**) was conducted on the included 23 studies, and excluding any one of the studies had no significant impact on the pooled effect value, which confirmed the stability of the final results of this study. The funnel chart showed absence of obvious publication bias (**Figure 4**).

In addition, twelve studies with a total of 2066 cases assessing detection positive rate of bacteria, fungi, viruses, and other pathogens, respectively. We introduced subgroups including bacteria, fungi, viruses, and other pathogens. In bacteria subgrop, their pooled result indicated that there was no difference in the mNGS group (55.97%, 553/988) and conventional methods group (41.93%, 452/1078) (*OR* = 1.73, 95% *CI* (0.73, 4.08), *P* = 0.21 and *I^2^* = 94%). The meta-analysis results showed that mNGS (17.75%, 163/973, 22.57%, 223/988 and 23.49%, 128/545) effectively increased the detection positive rate of fungi, virus, and other pathogens compared with the conventional methods group (8.44%, 89/1055) (*OR* = 2.04, 95% *CI* (1.13, 3.67), *P* = 0.02 and *I^2^* = 68%), (4.64%, 50/1078) (*OR* = 4.68, 95% *CI* (1.43, 15.39), *P* = 0.01 and *I^2^* = 82%) and (5.79%, 32/553) (*OR* = 2.59, 95% *CI* (1.68, 3.98), *P*=0.001 and *I^2^* = 72%) (**Figure S1**). The funnel chart indicated publication bias that are presented in **Figure S2**.

#### 28-day mortality

**Figure 5:**
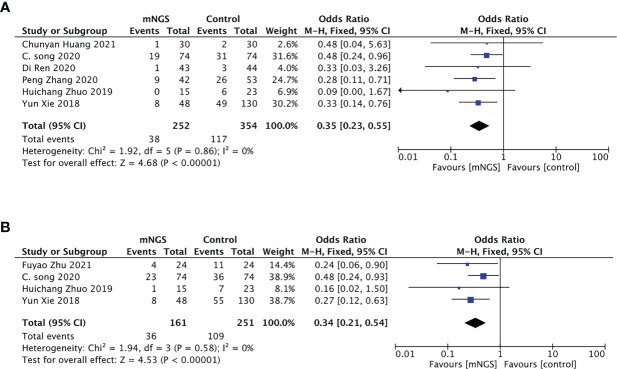
Primary outcome of the meta-analyses of mNGS compared with conventional methods group: **(A)**, 28-day mortality; **(B)**, 90-day mortality.

A total of six studies investigated the 28-day mortality. 252 cases were included in mNGS group, with a pooled mortality of 15.08% (38/252), significantly lower than that in the conventional methods group (33.05%, 117/354). The synthesis of these results derived from comparison with the conventional methods group indicated that mNGS can significantly promote patient survival, with an *OR* = 0.35, 95% *CI* (0.23, 0.55), *P* < 0.001, and *I^2^* = 0% (**Figure 5A**). The funnel chart indicated no obvious publication bias (**Figure S3**).

#### 90-day mortality

Four studies with 161 cases in mNGS group and 251 cases in the conventional methods group reported 90-day mortality. Adjusting treatment based on the pathogen detection results, the pooled 90-day mortality of the mNGS group was significantly lower than that in the conventional methods group (22.36%,36/161 vs 43.43%,109/251) (**Figure 5B**). There was no obvious publication bias (**Figure S4**).

#### Duration of mechanical ventilation

**Figure 6:**
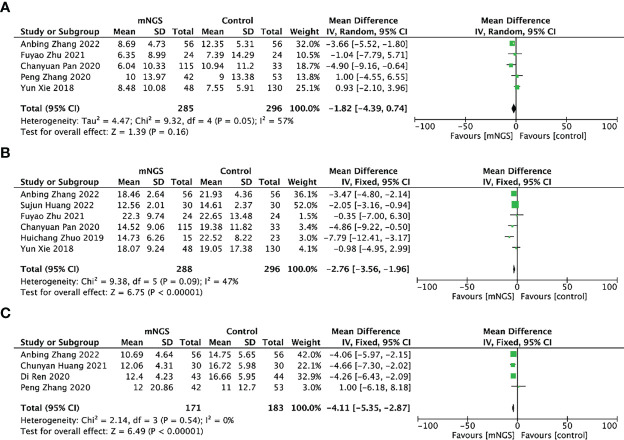
Secondary outcomes of the meta-analyses of mNGS compared with conventional methods group: **(A)**, duration of mechanical ventilation; **(B)**, length of hospital stay; **(C)**, length of stay in ICU.

In total, 5 studies including 285 cases in the mNGS group and 296 cases in the conventional methods group investigated the duration of mechanical ventilation. Their pooled result using random model indicated that there was no difference in the mNGS group and the conventional methods group (*MD* = − 1.82, 95% *CI* (− 4.39, 0.74), *P* = 0.16, *I^2^* = 57%) (**Figure 6A**). The funnel chart (**Figure S5**) showed no obvious publication bias.

#### Length of hospital stay

Six studies including 288 cases in the mNGS group and 296 cases in the conventional methods group reported the length of hospital stay. The mean length of hospital stay of the mNGS group was 16.33, shorter than that of the conventional methods group(19.74). The synthesized results proved that adjusting therapy according to mNGS results could reduce the length of hospital stay when compared with the conventional methods group, *MD* = -2.76, 95% *CI* [− 3.56, − 1.96], *P* < 0.00001, *I^2^* = 47% (**Figure 6B**). The funnel chart (**Figure S6**) showed absence of obvious publication bias.

#### Length of stay in ICU

Four studies including 171 cases in mNGS group and 183 cases in conventional methods group provided data on the length of stay in ICU. The mean length of stay in ICU of mNGS group and conventional methods group were 11.68 and 14.69. Patients who received mNGS detection had shorter length of stay in ICU than those receiving conventional methods detection [*MD* = -4.11, 95% *CI* (− 5.35, − 2.87), *P* < 0.001, *I^2^* = 0%] (**Figure 6C**). The funnel chart (**Figure S7**) indicated no obvious publication bias.

#### Antibiotic regiments changes based on NGS results

We carefully examined the studies included. The effects of mNGS results on antibiotic use were noted in four papers. Among them, Zhang et al. (Zhang et al., 2022) reported that early identification of pathogens through mNGS and timely adjustment of treatment regimens significantly reduced the frequency and duration of antimicrobial drug adjustment, which provides a new direction for antimicrobial drug management. Xu (Xu, 2021) and Chen (Chen, 2020) reported that adjusting antibiotic regimens based on mNGS detection results improved patient outcomes. Pan (Pan, 2020) reported that adjusted treatment based on mNGS results reduced length of hospital stay and duration of mechanical ventilation, and effectively reduced patient mortality.

## Discussion

Severe pneumonia is one of the most critical diseases with leading mortality among infectious diseases in ICU (Nair and Niederman, 2021). In the clinic, conventional methods sometimes fail to find the pathogen in patients with severe pneumonia. A reliable study (Chalmers et al., 2011) shows that the fatality rate is as high as 50% in patients who receive only empiric therapy without reliable evidence of pathogenic microorganisms. Prompt targeted antimicrobial therapy treatment is an effective means to improve the survival rate of patients with severe pneumonia.

The detection of DNA or RNA by mNGS provides rapid, efficient, and accurate access to the entire pathogen genome information within the whole test sample, which has been used widely for pathogen diagnosis in clinic in recent years (Miller et al., 2019). However, to our knowledge, there is no relevant systematic review and meta-analysis to provide more reliable evidence on the clinical values of mNGS on severe pneumonia. This study is performed to evaluate the diagnostic value and prognostic impact of mNGS compared with conventional methods in patients with severe pneumonia.

In order to accurately assess the pathogenic diagnostic value of mNGS in severe pneumonia and its impact on prognosis, we comprehensively collected the studies published on the application of mNGS to the etiological diagnostic of severe pneumonia compared with conventional methods, and finally included 23 studies for meta-analysis. The pathogen detection positive rate of the mNGS group was 80.48% (1233/1532), much higher than that of the conventional methods group (45.78%, 705/1540), which indicated that mNGS has a higher diagnostic value than conventional methods and can be used as an effective method for rapid etiological diagnosis of severe pneumonia. Bacteria can be detected by both mNGS and traditional detection methods. mNGS is superior for detecting fungi, viruses, and rare pathogens, which can be used as an adjunct or complementary tool to conventional microbial testing methods to provide an etiologic basis for the accurate anti-infection treatment of severe pneumonia. It is well known that the traditional microbial detection method is susceptible to the influence of normal bacteria in the body, especially after using antibiotics. Patients with severe pneumonia are often initially treated with an anti-microbial therapy, which reduces the positive rate of detection by conventional methods. mNGS can improve the pathogen detection positive rate and is less affected by external influences (Gu et al., 2019). Moreover, some reports have confirmed that mNGS achieve a faster diagnosis of pathogens and can detect unknown pathogens and even drug-resistance genes (Crofts et al., 2017). Another clinical application of mNGS is to identify microbial colonization or infection by monitoring the immune response of patients, ultimately achieving rational application of antibiotics, suppressing bacterial resistance, and reducing the economic and social burden of infectious diseases (Besser et al., 2018).

Meanwhile, compared with the conventional methods group, adjusting the treatment based on the mNGS results significantly decreased 28-day and 90-day mortality and shortened the length of hospital and ICU stay of patients with severe pneumonia. Patients with serious conditions have a short window of time for clinicians to save their lives and precise treatment is crucial to their prognosis. mNGS was faster, taking an average of two days, whereas conventional methods required at least three to five days (Xie et al., 2019). In addition, the higher pathogen-positive rate of mNGS detection gives clinicians the opportunity to select accurate anti-microbial drugs as early as possible, greatly increasing the proportion of target treatments. mNGS lead to more rapid required, accurate diagnosis than conventional methods in severe pneumonia. As a result, mNGS was associated with a better clinical prognosis of severe pneumonia.

mNGS facilitates accurate diagnosis and treatment. Still, there are many challenges, such as the lack of common standards for outcome analysis and common guidelines for report interpretation. The quality and stability of mNGS analysis results may not be stable to some extent, which is closely related to the operator’s skill and the detection efficiency of different laboratories may not be compared. A key challenge inherent to mNGS is that microbial nucleic acids from most patients’ samples are dominated by human host backgrounds (Hasan et al., 2016). Alternative methods exist for the depletion of human background DNA during the preanalytical phase, one approach is to selectively lyse human white blood cells using saponin or other chemical reagents (Marotz et al., 2018), and a different approach is to target low-molecular-weight cell-free DNA or RNA and remove high-molecular-weight genetic content that is often associated with human genomic material (Bukowska-Ośko et al., 2016). Another potential disadvantage of mNGS is the contamination of the sample. We need strictly adhere to quality control procedures for reagents and workflows to maintain as sterile and nucleic acid-free a testing environment as possible. The use of negative controls, reagent evaluation, and regular brushing is needed to ensure that laboratory and sample cross-contamination does not produce false positive results (Schlaberg et al., 2017). In addition, mNGS detection is highly sensitive and requires clinicians with rich experience to comprehensively consider the patient’s condition to make a judgment.

The included studies have limitations, such as small sample sizes, single-centered and retrospective analysis (Pan, 2020; Sun et al., 2020; Zhang et al., 2022), and confounding bias that could not be completely excluded. Besides, all 24 studies included were from China, making the inclusion of a homogeneous population and thus limiting the extensibility of the results. This limits the external generalizability of the results to some degree. Finally, we could not conduct a SORC curve analysis to assess the diagnostic efficacy of mNGS and conventional methods for severe pneumonia due to the scarcity of studies reporting sensitivity and specificity.

## Conclusion

The pathogen detection positive rate of mNGS was much higher than that of conventional methods, thereby indicating that mNGS has an extremely good diagnostic performance for severe pneumonia. Besides, adjusting treatment based on mNGS results can reduce the 28-day and 90-day mortality of patients with severe pneumonia, and shorten the length of hospital and ICU stay. Therefore, we suggest that patients with severe pneumonia should be tested for mNGS in addition to traditional culture as early as possible to improve the prognosis and reduce the length of hospital stay.

## Author contributions

We describe contributions to the paper as follows: conceptualization - ML, XD and GF. methodology - ML and XD. validation - ML and JY. formal analysis - ML and XD. data curation – ML, ChaZ and JY. writing original draft – ML. writing, review and editing - all listed. visualization - ML, XD and GF. supervision - XD and GF. All authors contributed to the article and approved the submitted version.
