# Supplementary material for: Clinical values of metagenomic next-generation sequencing in patients with severe pneumonia: a systematic review and meta-analysis
Source: Front Cell Infect Microbiol. 2023 Apr 6;13:1106859. doi: 10.3389/fcimb.2023.1106859 (PMC10117876; doi:10.3389/fcimb.2023.1106859)
Supplement: Supplementary file 1 [file DataSheet_1.docx]

Supplementary Material

**Clinical values of metagenomic next-generation sequencing**

**in patients with severe pneumonia: A systematic review and meta-analysis**

Autors: Minjie Lv, Changjun Zhu, Chenghua Zhu, Jing Yao, Lixu Xie, Changwen Zhang, Jianling Huang, Xingran Du, Ganzhu Feng

**Table of Contents:**

**Table S1** Search strategy in the databases.

**Table S2** The detection of bacteria, fungi, viruses, and other pathogens.

**Table S3** The quality score of the studies evaluated using the Newcastle-Ottawa Scale.

**Figure S1** Comparison of detection positive rate of different pathogens between mNGS and conventional methods group

**Figure S2** Funnel plot of detection positive rate of different pathogens between mNGS and conventional methods group

**Figure S3** Funnel plot of 28-day mortality between mNGS and conventional methods group.

**Figure S4** Funnel plot of 90-day mortality between mNGS and conventional methods group.

**Figure S5** Funnel plot of duration of mechanical ventilation between mNGS and conventional methods group.

**Figure S6** Funnel plot of length of hospital stay between mNGS and conventional methods group.

**Figure S7** Funnel plot of length of stay in ICU between mNGS and conventional methods group.

**Table S1** Search strategy in the databases

| Date of search performed:  28th September 2022. |
| --- |
| Databases searched:  PubMed, Embase, Cochrane Library, Web of Science, Clinical Trials.gov, CNKI, Wanfang DATA and CBM |
| Search terms:  "Next generation sequencing" [Mesh] OR "Metagenomic next generation sequencing" OR "NGS" OR "mNGS"  AND  "severe pneumonia" [Mesh] OR "serious pneumonia" [Mesh] OR "severe respiratory infection" [Mesh] OR "severe lung infection" [Mesh] OR "Severe community acquired pneumonia" [Mesh] OR "Severe hospital acquired pneumonia" [Mesh] |
| Restrictions:  None. |

| **Table S2** The detection of bacteria, fungi, virus and other pathogens | | | | | | | | | | | | |
| --- | --- | --- | --- | --- | --- | --- | --- | --- | --- | --- | --- | --- |
| **Author** | **Year** | **Bacteria** | |  | **Fungi** | |  | **Virus** | |  | **Other pathogens** | |
|  |  | mNGS, total(N) | Control, total(N) |  | mNGS, total(N) | Control, total(N) |  | mNGS, total(N) | Control, total(N) |  | mNGS, total(N) | Control, total(N) |
| Anbing Zhang | 2022 | 26,56 | 22,56 |  | 14,56 | 7,56 |  | 6,56 | 0,56 |  | 6,56 | 0,56 |
| Youlian Chen | 2022 | NR | NR |  | NR | NR |  | NR | NR |  | NR | NR |
| Xiaolian Zhou | 2022 | 17,70 | 23,70 |  | 3,70 | 12,70 |  | 2,70 | 0,70 |  | 47,70 | 9,70 |
| Sujun Huang | 2022 | NR | NR |  | NR | NR |  | NR | NR |  | NR | NR |
| Wenwen Tan | 2022 | NR | NR |  | NR | NR |  | NR | NR |  | NR | NR |
| Hanying Liu | 2022 | 92,173 | 158,173 |  | 30,173 | 16,173 |  | 15,173 | 0,173 |  | 31,173 | 0,173 |
| Chunyan Pan | 2021 | NR | NR |  | NR | NR |  | NR | NR |  | NR | NR |
| Fuyao Zhu | 2021 | NR | NR |  | NR | NR |  | NR | NR |  | NR | NR |
| Xiaolong Ma | 2021 | NR | NR |  | NR | NR |  | NR | NR |  | NR | NR |
| Jiancong Lu | 2021 | 40,76 | 27,76 |  | 11,76 | 2,76 |  | 6,76 | 2,76 |  | 11,76 | 8,76 |
| Jinlian Chen | 2021 | 10,20 | 4,20 |  | 5,20 | 3,20 |  | 2,20 | 3,20 |  | NR | NR |
| Yuhui Xu | 2021 | 40,110 | 32,110 |  | 35,110 | 13,110 |  | 22,110 | 10,110 |  | 7,110 | 5,110 |
| Peng Zhang | 2020 | 11,45 | 15,45 |  | 5,45 | 5,45 |  | 3,45 | 12,45 |  | 22,45 | 9,45 |
| Chanyuan Pan | 2020 | NR | NR |  | NR | NR |  | NR | NR |  | NR | NR |
| Di Ren | 2020 | NR | NR |  | NR | NR |  | NR | NR |  | NR | NR |
| C. Song | 2020 | NR | NR |  | NR | NR |  | NR | NR |  | NR | NR |
| Xiaodong Wu | 2020 | 252,329 | 95,329 |  | 41,329 | 8,329 |  | 148,329 | 0,329 |  | NR | NR |
| Ling Chen | 2020 | 10,28 | 8,28 |  | 7,28 | 3,28 |  | 9,28 | 0,28 |  | NR | NR |
| Xinyuan Fan | 2020 | NR | NR |  | NR | NR |  | NR | NR |  | NR | NR |
| Guoxian Sun | 2020 | NR | NR |  | NR | NR |  | NR | NR |  | NR | NR |
| Chunxi Pan | 2020 | NR | NR |  | NR | NR |  | NR | NR |  | NR | NR |
| Huichang Zhuo | 2019 | 13,15 | 4,23 |  | 0,15 | 0,23 |  | 2,15 | 1,23 |  | 4,15 | 1,23 |
| Jing Wu | 2019 | 9,18 | 5,18 |  | 9,18 | 2,18 |  | 7,18 | 1,18 |  | NR | NR |
| Yun Xie | 2018 | 33,48 | 59,130 |  | 3,48 | 18,130 |  | 11,48 | 21,130 |  | NR | NR |

NR: not reported

**Table S3** The quality score of the studies evaluated using the Newcastle-Ottawa Scale

| **Author** | **Year** | **Q1** | **Q2** | **Q3** | **Q4** | **Q5** | **Q6** | **Q7** | **Q8** | **Total** |
| --- | --- | --- | --- | --- | --- | --- | --- | --- | --- | --- |
| **Anbing Zhang** | 2022 | 1 | 1 | 1 | 1 | 2 | 1 | 0 | 0 | 7 |
| **Hanying Liu** | 2022 | 1 | 1 | 1 | 0 | 2 | 1 | 0 | 0 | 6 |
| **Sujun Huang** | 2022 | 1 | 1 | 1 | 1 | 2 | 1 | 0 | 0 | 7 |
| **Wenwen Tan** | 2022 | 1 | 1 | 1 | 0 | 2 | 1 | 0 | 0 | 6 |
| **Xiaolian Zhou** | 2022 | 1 | 1 | 0 | 0 | 2 | 1 | 0 | 0 | 5 |
| **Youlian Chen** | 2022 | 1 | 1 | 1 | 0 | 2 | 1 | 1 | 0 | 7 |
| **Chunyan Huang** | 2021 | 1 | 1 | 1 | 1 | 2 | 1 | 0 | 0 | 7 |
| **Fuyao Zhu** | 2021 | 1 | 1 | 1 | 0 | 2 | 1 | 1 | 0 | 7 |
| **Jiancong Lu** | 2021 | 1 | 1 | 0 | 0 | 2 | 1 | 0 | 0 | 5 |
| **Jinlian Chen** | 2021 | 1 | 1 | 1 | 1 | 2 | 1 | 0 | 0 | 7 |
| **Xiaolong Ma** | 2021 | 1 | 1 | 0 | 0 | 2 | 1 | 0 | 0 | 5 |
| **Yuhui Xu** | 2021 | 1 | 1 | 1 | 0 | 2 | 1 | 0 | 0 | 6 |
| **C.Song** | 2020 | 1 | 1 | 0 | 0 | 2 | 1 | 1 | 0 | 6 |
| **Chanyuan Pan** | 2020 | 1 | 1 | 0 | 1 | 2 | 1 | 0 | 0 | 6 |
| **Chunxi Pan** | 2020 | 1 | 1 | 1 | 1 | 2 | 1 | 0 | 0 | 7 |
| **Di Ren** | 2020 | 1 | 1 | 0 | 1 | 2 | 1 | 0 | 0 | 6 |
| **Guoxian Sun** | 2020 | 1 | 1 | 1 | 0 | 2 | 1 | 1 | 0 | 7 |
| **Ling Chen** | 2020 | 1 | 1 | 1 | 0 | 2 | 1 | 0 | 0 | 6 |
| **Peng Zhang** | 2020 | 1 | 1 | 0 | 0 | 2 | 1 | 0 | 0 | 5 |
| **Xiaodong Wu** | 2020 | 1 | 1 | 1 | 1 | 2 | 1 | 1 | 0 | 8 |
| **Xinyuan Fan** | 2020 | 1 | 1 | 1 | 0 | 2 | 1 | 0 | 0 | 6 |
| **Huichang Zhuo** | 2019 | 1 | 1 | 1 | 1 | 2 | 1 | 1 | 0 | 8 |
| **Jing Wu** | 2019 | 1 | 1 | 1 | 0 | 2 | 1 | 1 | 0 | 7 |
| **Yun Xie** | 2018 | 1 | 1 | 0 | 0 | 2 | 1 | 1 | 0 | 6 |

**Table S3** The quality score of the studies evaluated using the Newcastle-Ottawa Scale*（Continued）*

| **Footnotes：**  **Modified Newcastle – Ottawa Quality Assessment Scale (NOS)** |
| --- |
| CASE CONTROL STUDIES |
| **Selection**  **1) Is the case definition adequate?**   1. **yes, with some independent validation (e.g. >1 person/record/time/process to extract information, or reference to primary record source such as x-rays or medical/hospital records) *** 2. **yes, e.g., record linkage (e.g. ICD codes in database) or self-report with no reference to primary record** 3. **no description**   **2) Representativeness of the cases**   1. **all eligible cases with outcome of interest over a defined period of time, all cases in a defined catchment area, all cases in a defined hospital or clinic, group of hospitals, health maintenance organisation, or an appropriate sample of those cases (e.g. random sample) *** 2. **not satisfying requirements in part (a), or not stated**   **3) Selection of controls**   1. **community controls (i.e. same community as cases and would be cases if had outcome) *** 2. **hospital controls, within same community as cases (i.e. not another city) but derived from a hospitalised population** 3. **no description**   **4) Definition of controls**   1. **it must explicitly state that controls have no history of this outcome *** 2. **no description** |
| **Comparability**  **1) Comparability of cases and controls on the basis of the design or analysis**   1. **study controls for one factor *** 2. **study controls for any additional factor *** |
| **Exposure**  **1) Ascertainment of exposure**   1. **secure record (e.g., medical records) *** 2. **structured interview where blind to case/control status *** 3. **interview not blinded to case/control status** 4. **written self report or medical record only** 5. **no description**   **2) Same method of ascertainment for cases and controls**   1. **yes *** 2. **no**   **3) Non-Response rate**   1. **same rate for both groups *** 2. **non respondents described** 3. **rate different and no designation** |
| **COHORT STUDIES** |
| **Selection**  **1) Representativeness of the exposed cohort**   1. **truly representative of the average in the community (>75% catchment population) *** 2. **somewhat representative of the average in the community (<75% catchment population) *** 3. **selected group of users e.g., nurses, volunteers** 4. **no description of the derivation of the cohort**   **2) Selection of the non exposed cohort**   1. **drawn from the same community as the exposed cohort *** 2. **drawn from a different source** 3. **no description of the derivation of the non exposed cohort**   **3) Ascertainment of exposure**   1. **secure record (eg medical records) *** 2. **structured interview *** 3. **written self report** 4. **no description**   **4) Demonstration that outcome of interest was not present at start of study**   1. **yes *** 2. **no** |
| **Comparability**  **1) Comparability of cohorts on the basis of the design or analysis**   1. **study controls for one factor *** 2. **study controls for additional factors *** |
| **Outcome**  **1) Assessment of outcome**   1. **capture- recapture *** 2. **clinical or laboratory record linkage *** 3. **self report** 4. **no description**   **2) Was follow-up long enough for outcomes to occur**   1. **yes *** 2. **no**   **3) Adequacy of follow up of cohorts**   1. **all cases reported *** 2. **cases not reported unlikely to introduce bias- > 75% cases reported *** 3. **< 75% cases reported** 4. **no statement** |


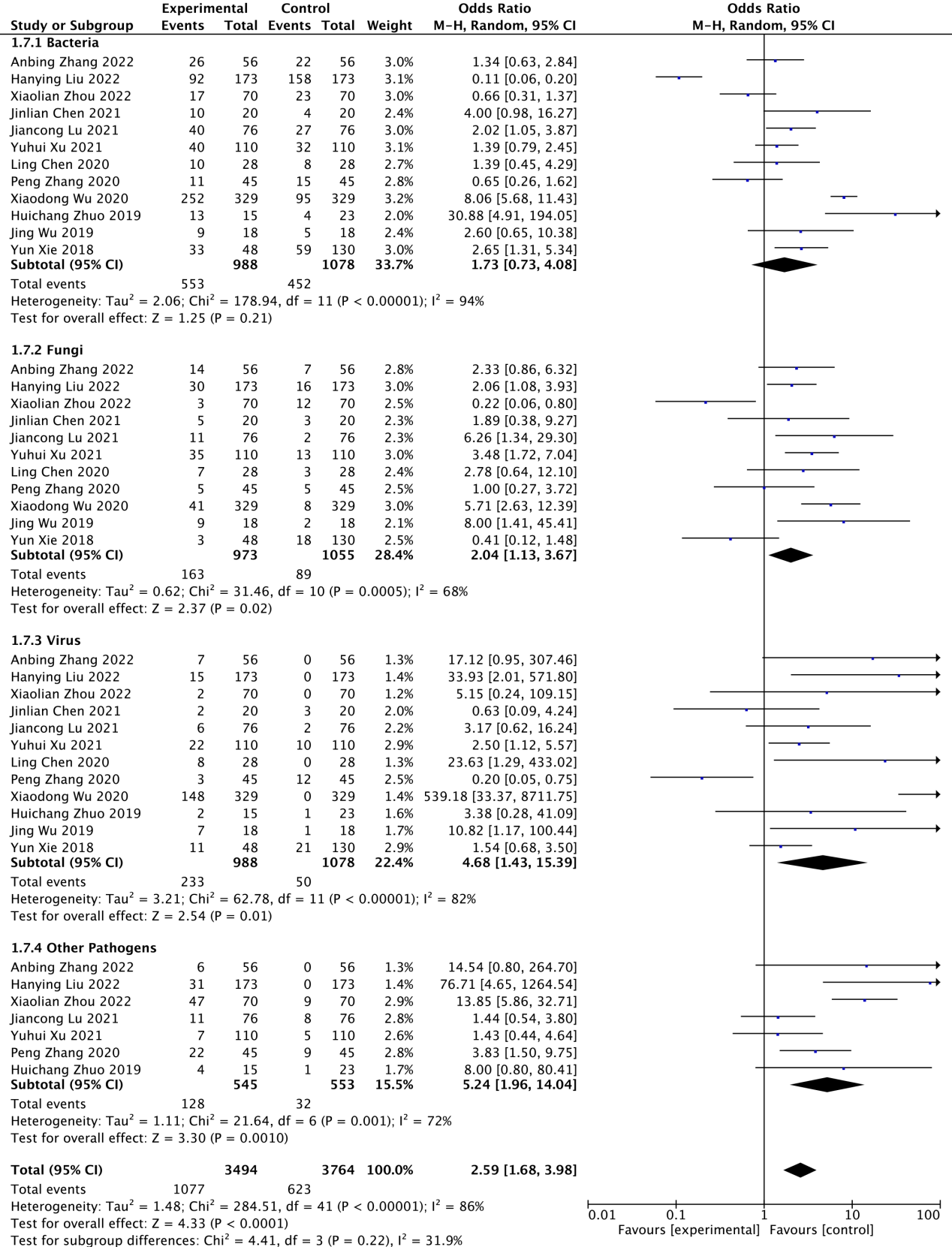


**Figure S1** Comparison of detection positive rate of different pathogens between mNGS and conventional methods group


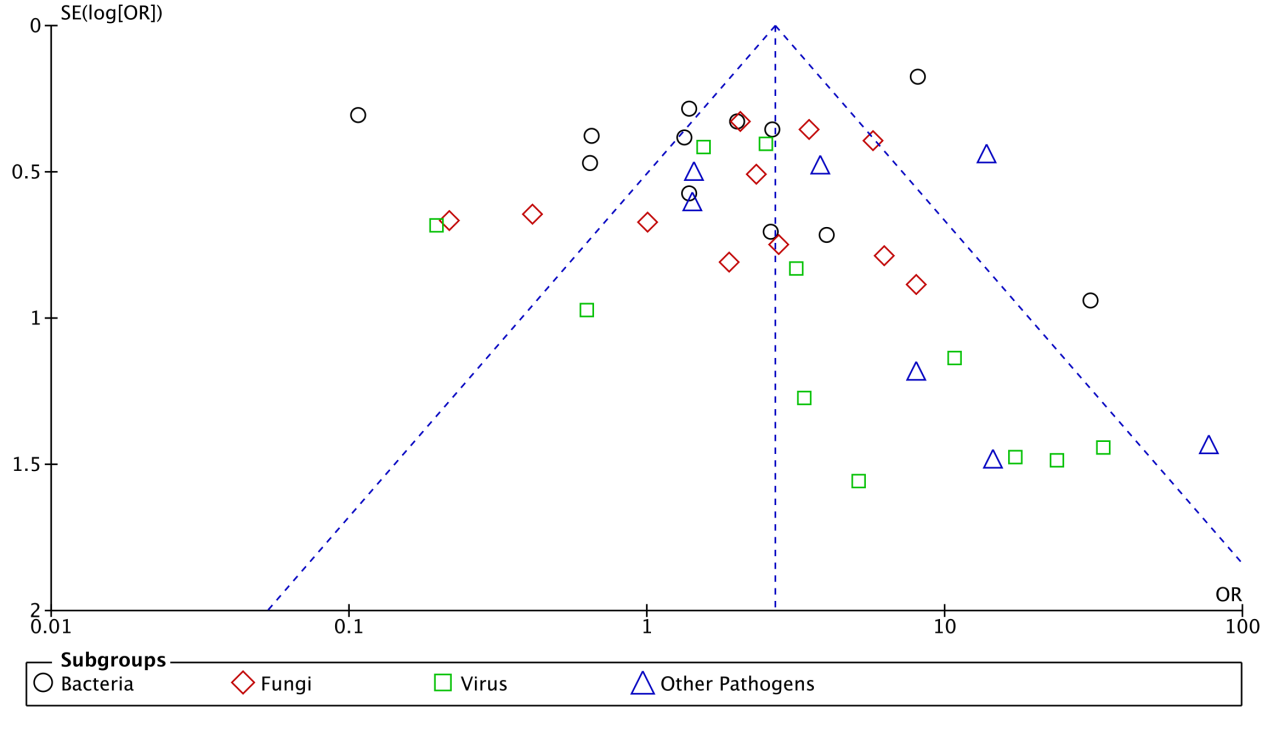


**Figure S2** Funnel plot of detection positive rate of different pathogens between mNGS and conventional methods group


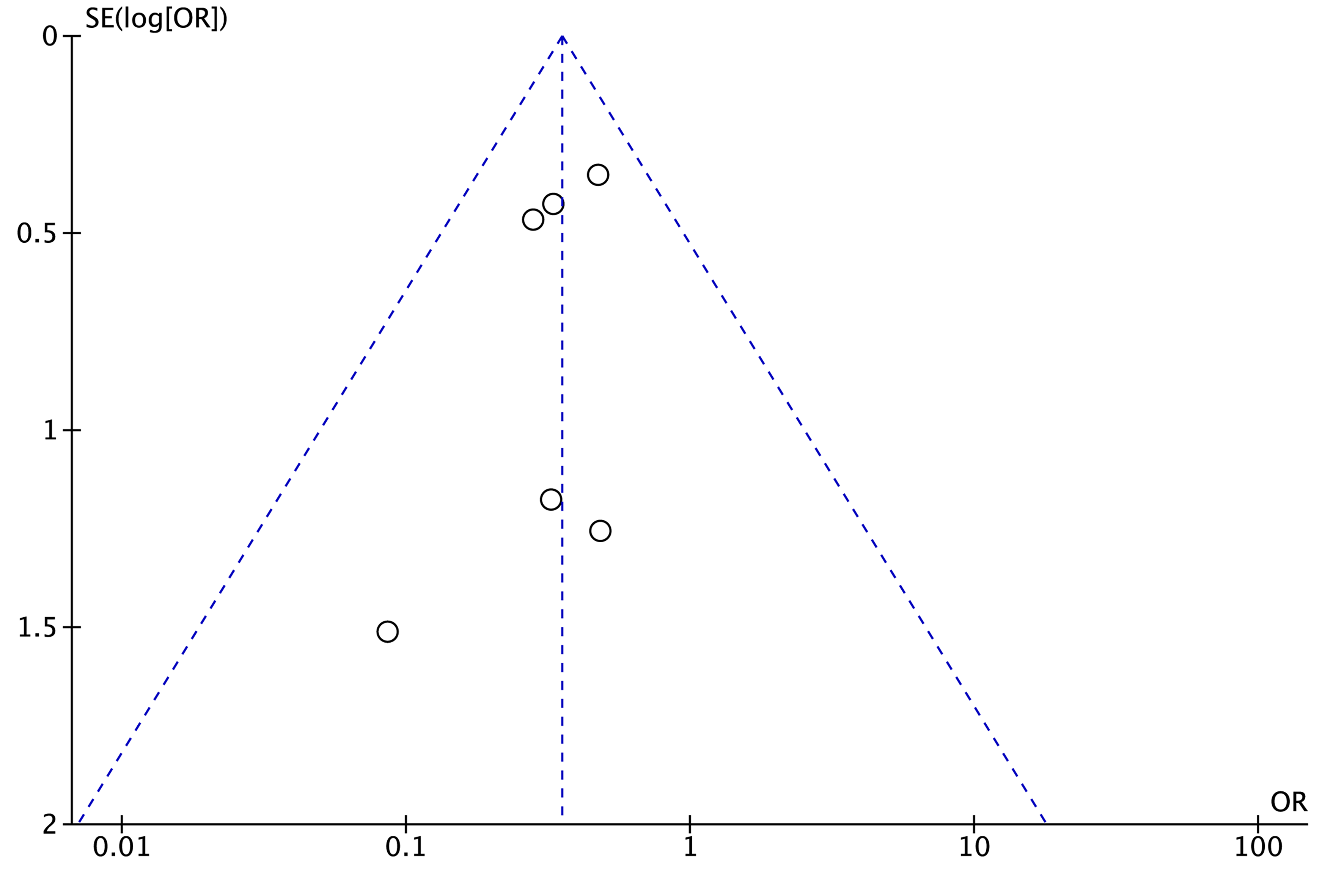


**Figure S3** Funnel plot of 28-day mortality between mNGS and conventional methods group.


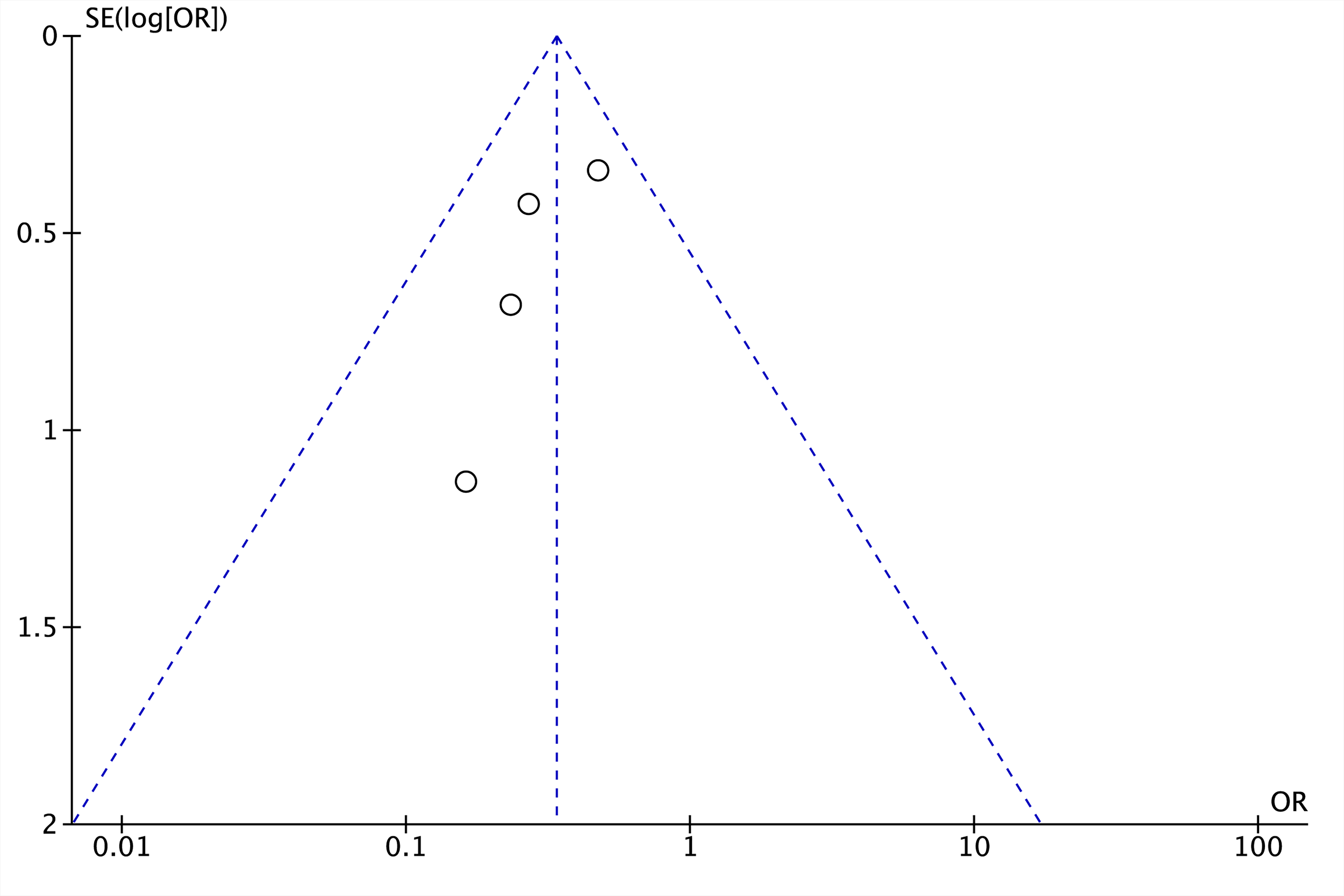


**Figure S4** Funnel plot of 90-day mortality between mNGS and conventional methods group.


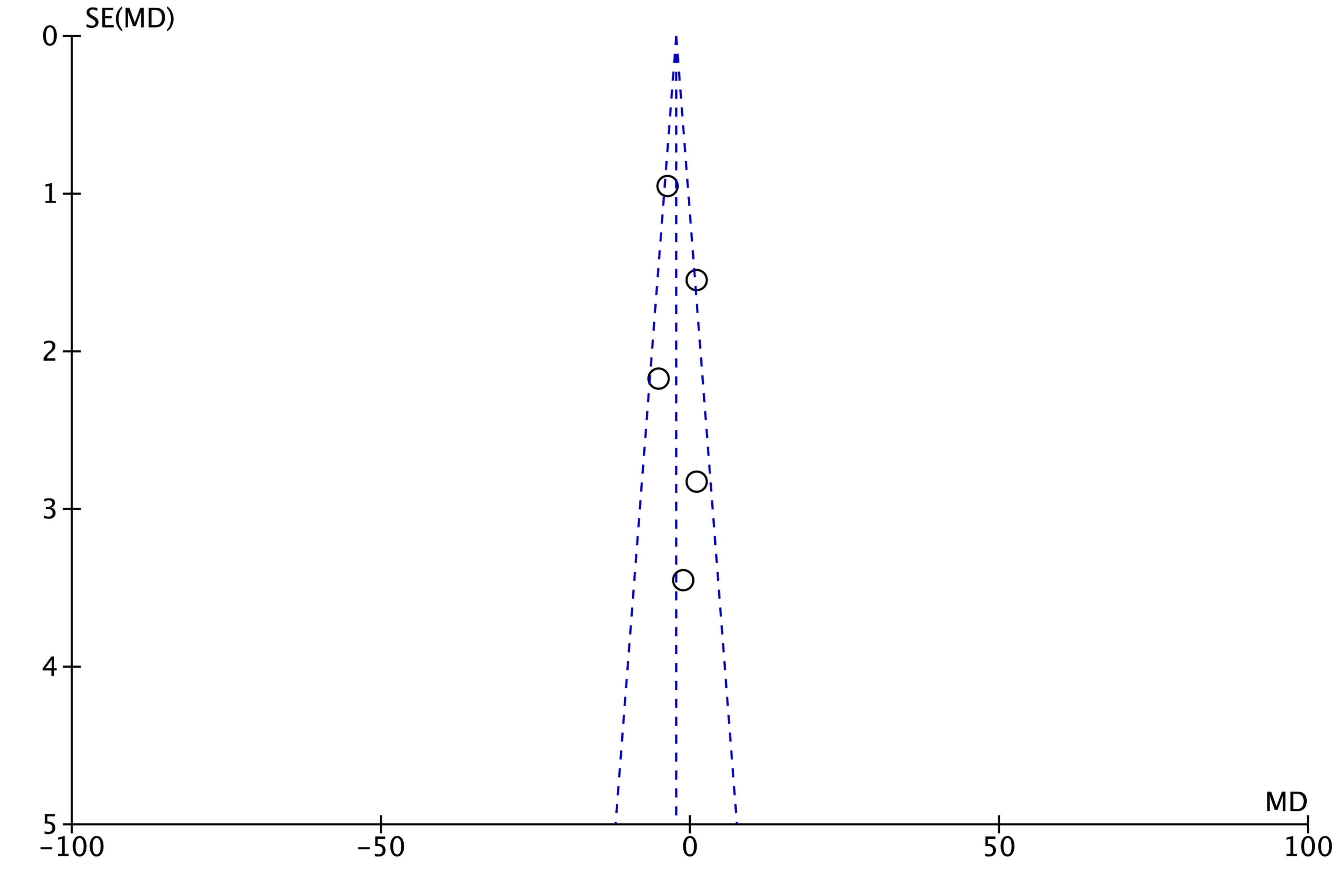


**Figure S5** Funnel plot of duration of mechanical ventilation between mNGS and conventional methods group.


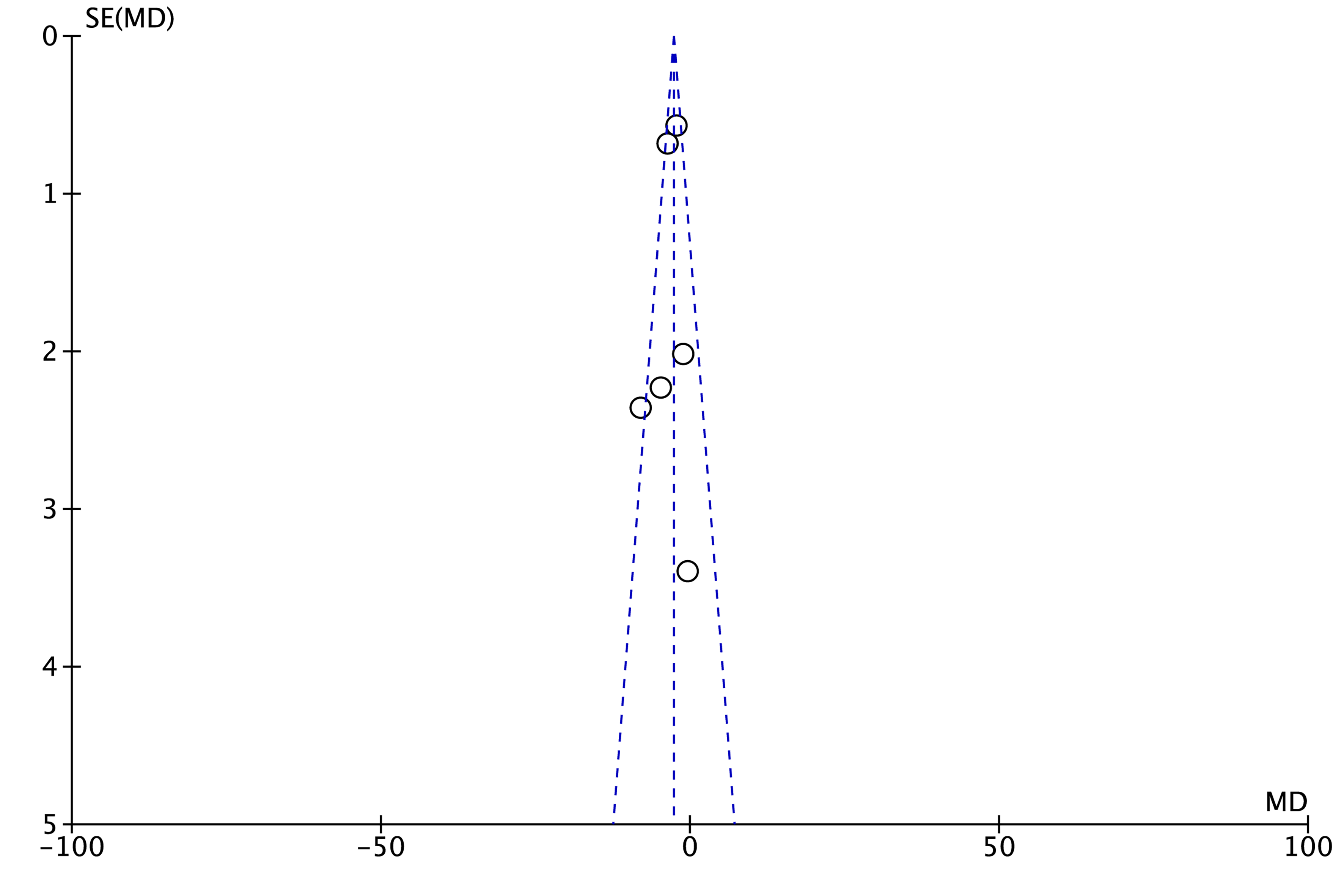


**Figure S6** Funnel plot of length of hospital stay between mNGS and conventional methods group.


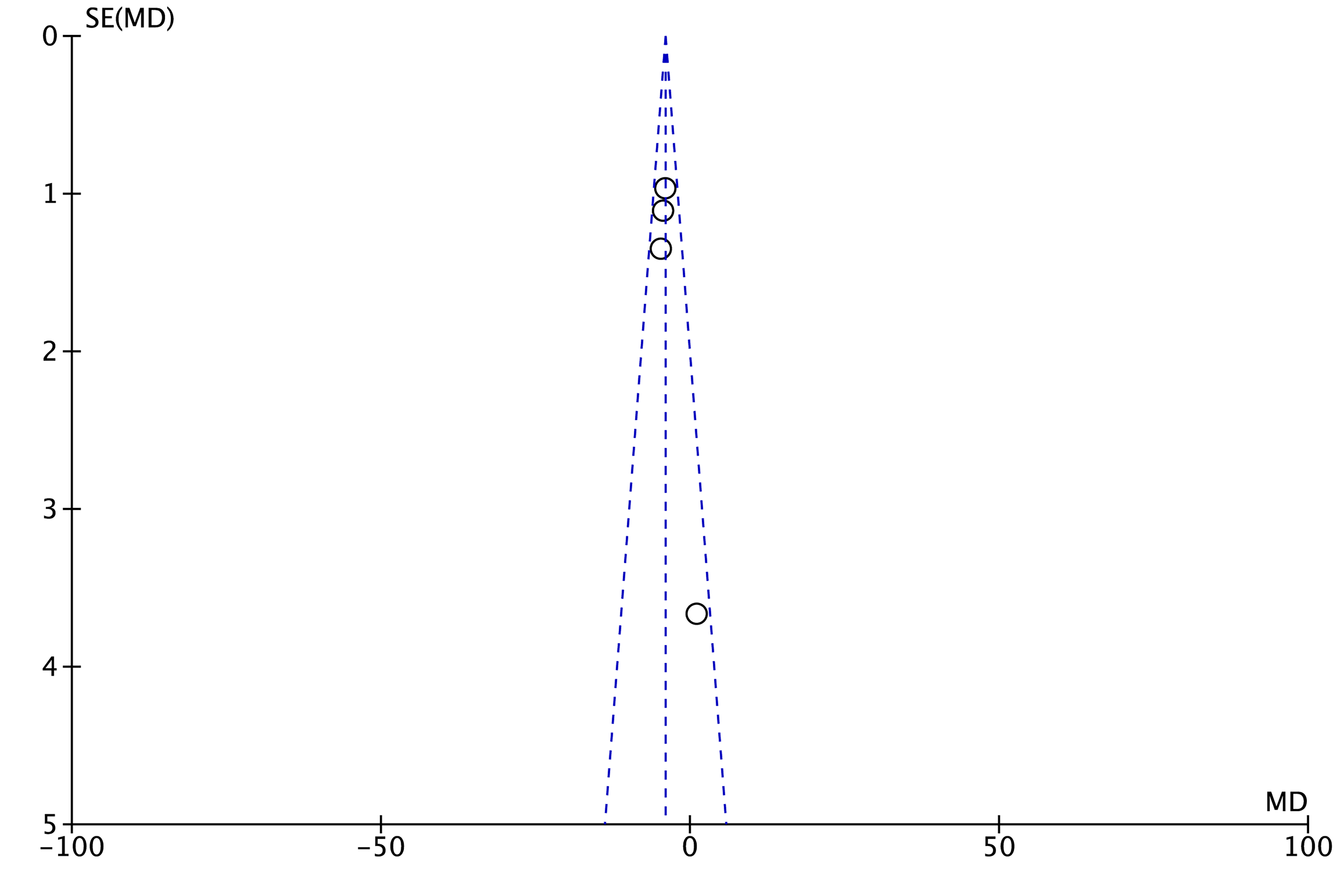


**Figure S7** Funnel plot of length of stay in ICU between mNGS and conventional methods group.
